# Trichostatin A Inhibits Epithelial Mesenchymal Transition Induced by TGF-β1 in Airway Epithelium

**DOI:** 10.1371/journal.pone.0162058

**Published:** 2016-08-29

**Authors:** Il-Ho Park, Ju-Hyung Kang, Jae-Min Shin, Heung-Man Lee

**Affiliations:** 1 Department of Otorhinolaryngology-Head and Neck Surgery, Guro Hospital, Korea University College of Medicine, Seoul, South Korea; 2 Department of Biomedical Sciences, Korea University Graduate School, Seoul, South Korea; 3 Medical Devices support Center, Guro Hospital, Korea University College of Medicine, Seoul, South Korea; Indiana University School of Medicine, UNITED STATES

## Abstract

**Background and Objectives:**

Tissue remodeling is believed to cause recalcitrant chronic rhinosinusitis (CRS). Epithelial-mesenchymal transition (EMT) is a novel clinical therapeutic target in many chronic airway diseases related with tissue remodeling. The aim of this study was to investigate the effect of trichostatin A (TSA) on transforming growth factor (TGF)-β1-induced EMT in airway epithelium and nasal tissue.

**Materials and Methods:**

A549 cells, primary nasal epithelial cells (PNECs), or inferior nasal turbinate organ culture were exposed to TSA prior to stimulation with TGF-β1. Expression levels of E-cadherin, vimentin, fibronectin, α-smooth muscle actin (SMA), histone deacetylase 2 (HDAC2), and HDAC4 were determined by western blotting and/or immunofluorescent staining. Hyperacetylation of histone H2 and H4 by TSA was measured by western blotting. After siHDAC transfection, the effects of HDAC2 and HDAC4 silencing on expression of E-cadherin, vimentin, fibronectin, α-SMA, HDAC2, and HDAC4 in TGF-β1-induced A549 were determined by RT-PCR and/or western blotting. We assessed the change in migration capacity of A549 cells by using cell migration assay and transwell invasion assay.

**Results:**

TGF-β1 altered mRNA and protein expression levels of EMT markers including E-cadherin, vimentin, fibronectin, α-SMA, slug, and snail in A549 cells. Inhibition and silencing of HDAC2 and HDAC4 by TSA and siRNA enhanced TGF-β1-induced EMT in A549 cells. TSA blocked the effect of TGF-β1 on the migratory ability of A549 cells. In experiments using PNECs and inferior turbinate organ cultures, TSA suppressed expression of EMT markers induced by TGF-β1.

**Conclusions:**

We showed that EMT is induced by TGF-β1 in airway epithelial cells and nasal tissue via activation of HDAC2 and HDAC4, and that inhibition of HDAC2 and HDAC4 by TSA reduces TGF-β1-induced EMT. This observation indicates that histone deacetylase inhibitors such as TSA could be potential candidates for treatment of recalcitrant CRS related with tissue remodeling.

## Introduction

Chronic rhinosinusitis (CRS) is an inflammation of the nose and paranasal sinuses characterized by nasal blockage, nasal discharge, and olfactory dysfunction lasting more than 12 weeks [1]. Control of CRS can be defined as a resolution of symptoms combined with the recovery of mucosa. Medical treatment options for CRS include oral antibiotics, topical corticosteroids, systemic steroids, and other medications such as antihistamines, mucolytics, and decongestants. It is known that about one-third of the symptoms of CRS are relieved by medical treatment [2]. Endoscopic sinus surgery is considered an option after failure of above medical treatment. However, the disease persists in one-third of patients one year after surgery [3]. In spite of considerable effort to identify factors related to disease recalcitrance, such factors are still not clearly understood.

Epithelial-mesenchymal transition (EMT) is a phenotype conversion that turns a polarized epithelial cell into a mesenchymal cell. In the process of EMT, epithelial cells lose cell-to-cell adhesion and apical-basal polarity, reorganize their cytoskeletal protein, and acquire the characteristics of mesenchymal cells, such as enhanced motility, invasiveness, and fibrogenesis [4,5]. EMT is known as a feature of embryogenesis, organ development, and cancer progression [6]. It is also activated in wound healing and inflammation, and dysregulation of EMT by repeated stress caused by them may lead to organ fibrosis [7,8]. Additionally, evidence has shown that CRS is related to EMT [9,10].

In a previous study, we showed that histone deacetylase (HDAC) inhibition by trichostatin A (TSA) is associated with extracellular matrix accumulation in nasal polyp-derived fibroblasts [11]. As extracellular matrix accumulation is one of the main features of mesenchymal cells, we hypothesized that epigenetic regulation by TSA can also be associated with suppression EMT of airway epithelium. The purposes of this study were to investigate whether EMT is induced by activation of HDACs in airway epithelial cells and nasal tissue, and to evaluate the effect that histone deacetylase inhibitors such as TSA have on EMT. We stimulated cells and tissues with transforming growth factor (TGF)-β1, which is known to induce EMT, according to several studies [12,13].

## Materials and Methods

### Materials

Human recombinant TGF-β1 was obtained from R&D Systems (Minneapolis, MN). TSA was purchased from Sigma (St. Louis, MO, USA). Cells or tissues were previously exposed to TGF-β1 (5mg/mL) after pretreatment for 1 hour with TSA (100nM)

### Cell culture

A549 (human carcinomic alveolar basal epithelial cells, type II) cells were obtained from the American Type Culture Collection (Manassas, VA). A549 cells were grown in RPMI-1640 medium containing 10% (v/v) heat-inactivated fetal bovine serum (Invitrogen, Carlsbad, CA), 1,000 unit/mL penicillin, and 1,000 μg/mL streptomycin (Invitrogen).

Inferior turbinate mucosa specimens were obtained from six patients during endoscopic sinus surgery for benign tumors at the Department of Otorhinolaryngology, Korea University Medical Center. None of the patients had a history of allergies, asthma, or aspirin sensitivity, nor had any of them received steroids, nonsteroidal anti-inflammatory drugs, antihistamines, or antibiotics during the 4 weeks prior to the biopsy. For the primary culture of the nasal epithelial cells, the nasal tissues were washed with phosphate buffered saline and immersed in Dispase (Stem cell technologies, Vancouver, Canada) for 4 h. Then, the tissue was filtered through a mesh. Primary nasal epithelial cells (PNECs) were incubated with Bronchial Epithelial Cell Growth Medium (Lonza, Basel, Switzerland). Written informed consent was obtained from each patient, and the study was approved by the Korea University Medical Center Institutional Review Board (KUGGR-12041-001).

### Organ culture of nasal polyps

Inferior turbinates from the patients were cut into three pieces of 2 to 3 mm with scissors under sterile conditions. Tissue fragments were washed three times with phosphate buffered saline. The washed tissue fragments were placed on a prehydrated gelatin sponge (10 mm × 10 mm × 1 mm; Spongostan, Johnson & Johnson, San Angelo, TX) in 6-well plates. Then, each well was filled with 1.5 mL of culture medium containing Dulbecco’s Modified Eagle Medium (Invitrogen) supplemented with 2% fetal bovine serum (Invitrogen). Inferior turbinate tissues were stimulated with TGF-β1 (5 ng/mL) with or without TSA. The plates were maintained at 37°C in 5% CO2.

### 3-(4,5-Dimethylthiazol-2-yl)-2,5-diphenyl tetrazolium bromide assay

A549 cells were seeded on 96-well tissue culture plates at a concentration of 4 x 10^5^ cells/mL with various concentrations (0–1600 nM) of TSA with or without TGF-β1 (5 ng/mL) for 72 h. Then, cells were incubated with MTT (3-(4,5-dimethylthiazol-2-yl)-2,5-diphenyl tetrazolium bromide, Sigma) for 4 h, and the reaction was interrupted by the addition of acidified isopropanol. A fluorescence microplate reader (F2000; Hitachi, Ltd., Tokyo, Japan) was used to determine the results (570 nm).

### Immunofluorescent staining

Cells were incubated with TGF-β1 (5 ng/mL) alone or in conjunction with TSA for 72 h. Images were obtained with a microscope (Olympus BX51; Olympus, Tokyo, Japan). Cells were fixed with 4% paraformaldehyde, then permeabilized with 0.2% TritonX-100 in 1% bovine serum albumin for 10 min, blocked with 5% bovine serum albumin for 1 h at room temperature, and incubated overnight at 4°C with monoclonal antibodies including vimentin, α-SMA, and snail, and polyclonal antibodies including E-cadherin, fibronectin, and slug (Santa Cruz, CA). Cells were then incubated with Dy-Light 549 horse anti-mouse IgG antibody or DyLight 488 horse anti-rabbit IgG Antibody (Vector Labs, Burlingame, CA). Finally, cells were counterstained with 4’,6-diamidino-2-phenylindole (Invitrogen, Carlsbad, CA). Immunostained cells were captured and visualized using a confocal microscope (LSM700; Zeiss, Oberkochen, Germany).

### Reverse transcription-polymerase chain reaction (RT-PCR)

Total RNA was isolated according to the manufacturer’s recommendations using Trizol reagent (Invitrogen). Two micrograms of RNA were reverse-transcribed using MMLV reverse transcriptase (Invitrogen) according to the manufacturer’s protocol. PCR was performed using the following primers: HDAC2 (sense sequence 5′- CATCCCATGAAGCCTCATAGAATC -3′, anti-sense sequence 5′- GCACCAATATCCCTCAAGTCTCC -3′, 566 bp), HDAC4 (sense sequence 5′- CTG CAAGTGGCCCCCTCGG -3′, anti-sense sequence 5′- CTCGTGCTGTTGCCTCTGGA -3′, 179 bp), Snail (sense sequence 5′- TCTAGGCCCTGGCTGCTACAA -3′, anti-sense sequence 5′- GCCTGGCACTGGTACTTCTTGAC -3′, 152 bp), Slug (sense sequence 5′- ATGCATATTCGGACCCACACATTA -3′, anti-sense sequence 5′- AGAATTTGACCTGTCTGCAAATGCT -3′, 158 bp), and GAPDH (sense sequence 5′- GTGGATATTGTTGCCATCAATGACC -3′, anti-sense sequence 5′- GCCCCAGCCTTCTTCATGGTGGT -3′, 271 bp). The gels were captured and visualized using Molecular Imager ChemiDoc XRS+ (Bio-Rad, Hercules, CA).

### Western blot analysis

A549 cells were lysed in PRO-PREPTM protein extraction solution (iNtRON Biotechnology, Seongnam, Korea). Lysates were separated by 10% sodium dodecyl sulfate polyacrylamide gel electrophoresis and transferred onto polyvinyl difluoride membranes (Millipore Inc., Billerica, MA). Membranes were blocked with a 5% skim milk solution and incubated with the following antibodies: E-cadherin, vimentin, α-SMA, fibronectin, snail, slug (Santa Cruz, CA), HDAC2, HDAC4, ac-histone H3, histone H3, ac-histone H4, histone H4 (Upstate, Millipore Inc.), and β-actin (Santa Cruz, CA). The blots were visualized with HRP-conjugated secondary antibodies and an ECL system (Pierce, Rockford, IL).

### Transfection with small interference RNA (siRNA) of HDAC2 and HDAC4

A549 cells were pelleted by centrifugation at 13,000 rpm for 3 min; thereafter, the cells were suspended in 1 mL phosphate buffered saline and dispersed using a pipette. The cells were pelleted at 1,000 rpm for 1 min. The supernatant was discarded, and the cells were suspended in Neon Resuspension buffer (Invitrogen) at a concentration of 6 × 10^5^ cells/mL. Universal negative control siRNA (siControl; Santa Cruz) and small interference oligonucleotide RNA directed against siHDAC2 and siHDAC4 (Santa Cruz) were used as controls. Neon Electrolytic buffer (Invitrogen) was added into the Neon transfection tubes, and the tubes were then placed in the Neon transfection system device (Invitrogen) that was set to 1400 V and 30 pulses. Gold tips were used to aspirate 100 μL RNA cell mixture and place it in the device station. After electroporation, an appropriate amount of complete medium was immediately added to each cell aliquot, and the cells were re-plated onto culture dishes.

### Cell migration scratch assays

A549 cells were plated and grown to confluence in 6-well tissue culture dishes. A straight scratch was made in the cells using a pipette tip. Scratched cells were immediately rinsed with phosphate buffered saline, and RPMI-1640 medium containing 10% (v/v) heat-inactivated fetal bovine serum (Invitrogen, Carlsbad, CA, USA), 1,000 unit/mL penicillin, and 1,000 μg/mL streptomycin (Invitrogen) was added. Cells were incubated with TGF-β1 (5 ng/mL) alone or in conjunction with TSA for 48 h. Images were obtained with a microscope (Olympus BX51; Olympus, Tokyo, Japan).

### Transwell migration assay

The cells were seeded to the upper chamber of transwell chambers (Corning Life Sciences, MA, USA). Then, RPMI-1640 medium containing 10% (v/v) heat-inactivated fetal bovine serum (Invitrogen, Carlsbad, CA, USA), 1,000 unit/mL penicillin, and 1,000 μg/mL streptomycin (Invitrogen) was treated with TGF-β1 (5 ng/mL) alone or in conjunction with TSA to the lower chamber of transwell chambers for 48 h. The cells on the upper surface of the membrane were removed by cotton swabs. Then, the cells on the lower surface of the membrane were stained with Diff-Quik stain (Sysmex, Kobe, Japan). Images of the stained cells from five selected views were captured under a microscope at 400x magnification.

### Statistical analysis

Results were obtained from at least three independent experiments. The statistical significance of the differences between control and experimental data was analyzed with unpaired two-way analysis of variance (ANOVA) test or one-way ANOVA followed by Tukey’s test (GraphPad Prism, version 5, Graph Pad Software, San Diego, CA). Significance was established at the 95% confidence level; *p* values less than 0.05 were accepted as statistically significant.

## Results

### TSA inhibits TGF-β1-induced EMT in A549 cells

**Fig 1:**
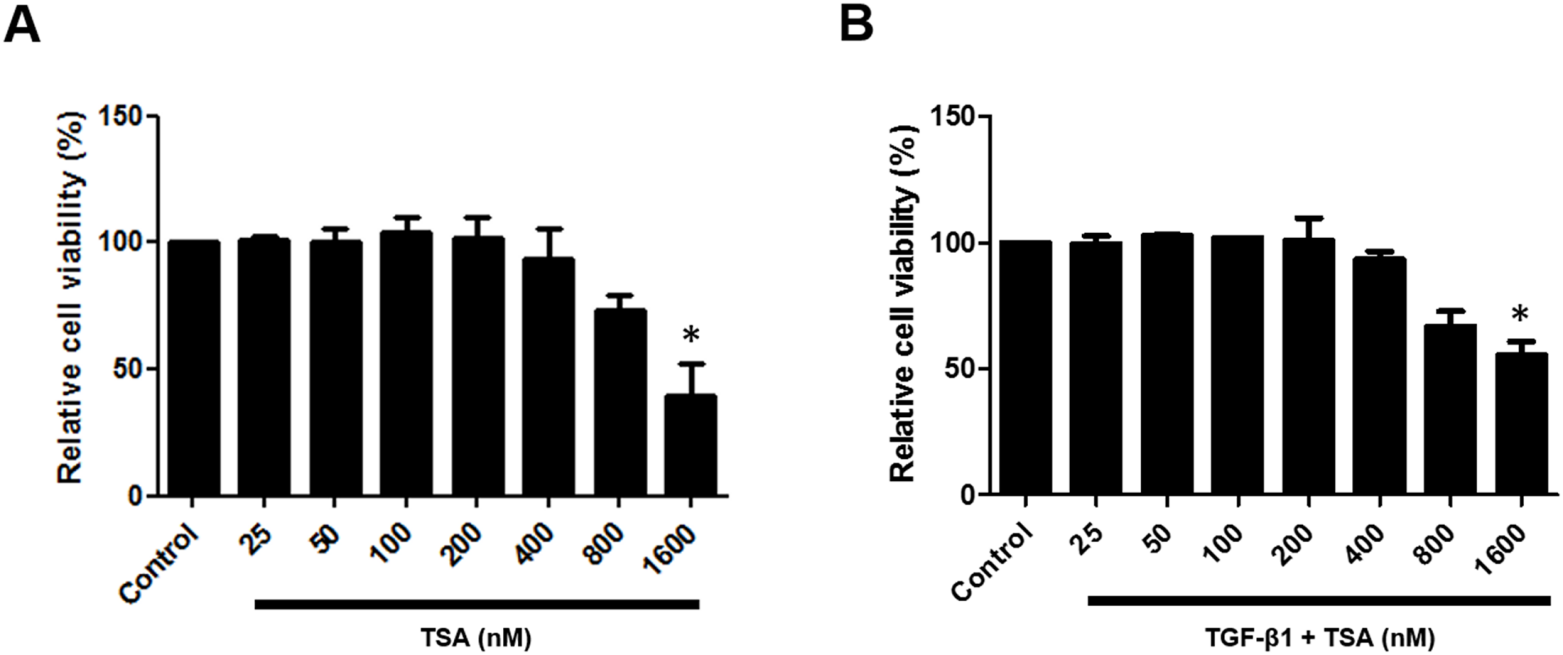
Cytotoxicity of histamine determined by MTT assay. MTT, 3-(4,5-dimethylthiazol-2yl)-2,5-diphenyl tetrazolium bromide, *P < 0.05 vs. control.

An MTT assay was performed to examine the effects of TSA on survival of A549 cells. Serial dilutions of A549 cells and MTT reagent were used to generate a cell titration curve. The standard curve indicated a linear relationship between number of cells and absorption at 570 nm. Concentrations of TSA ranging from 0 to 1600 nM were examined. TSA did not affect cell survival at concentrations below 400 nM regardless of the presence of TGF- β1 (Fig 1).

**Fig 2:**
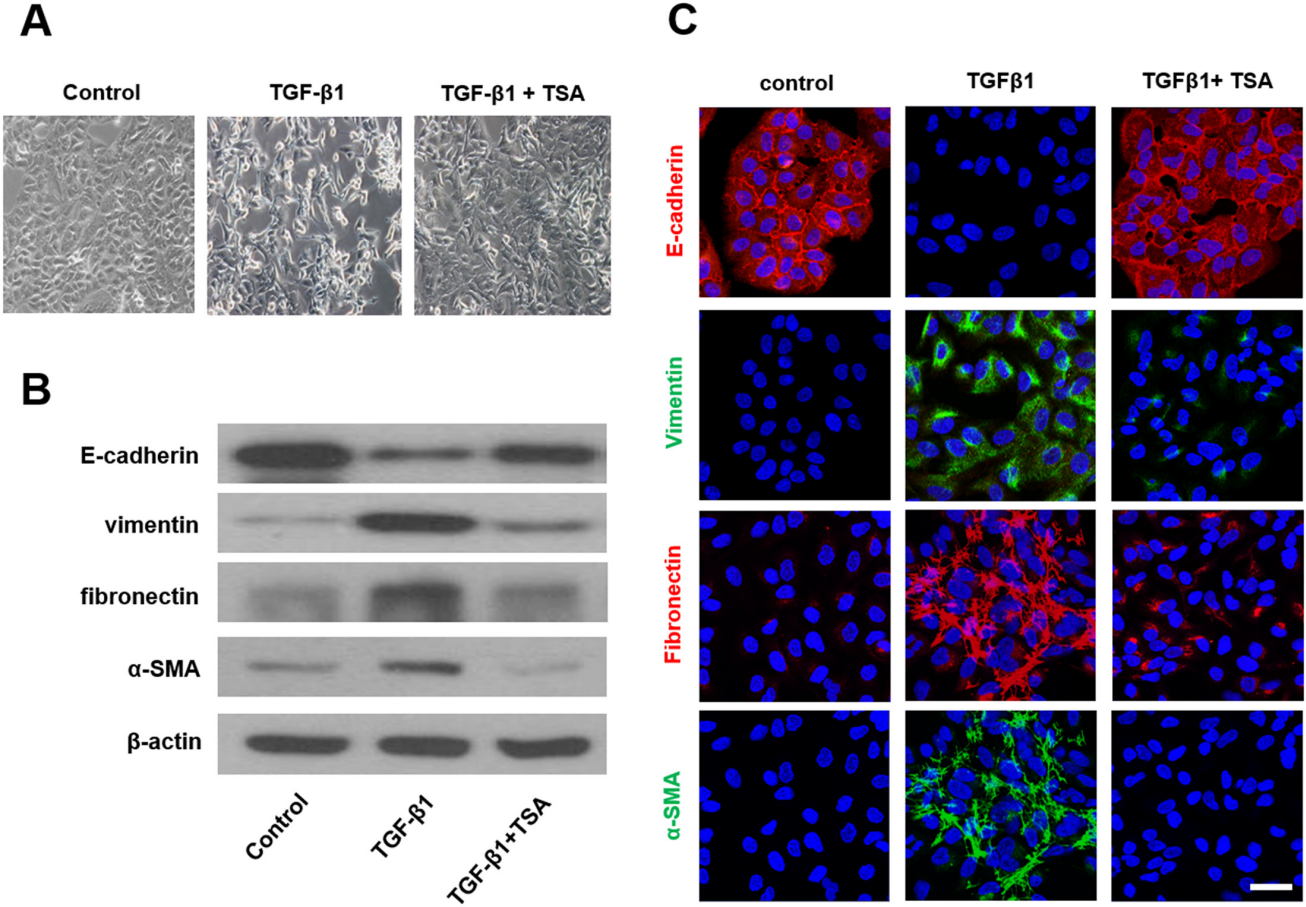
(A) Effects of trichostatin A on morphology of TGF-β1-stimulated A549 cells as observed under a phase contrast microscope. Effects of trichostatin A on expression of E-cadherin, vimentin, fibronectin, and α-smooth muscle actin protein in TGF-β1-stimulated A549 cells were determined by western blotting (B) and immunofluorescent staining (C). Representative of independent experiments. Scale bar = 50 μm.

**Fig 3:**
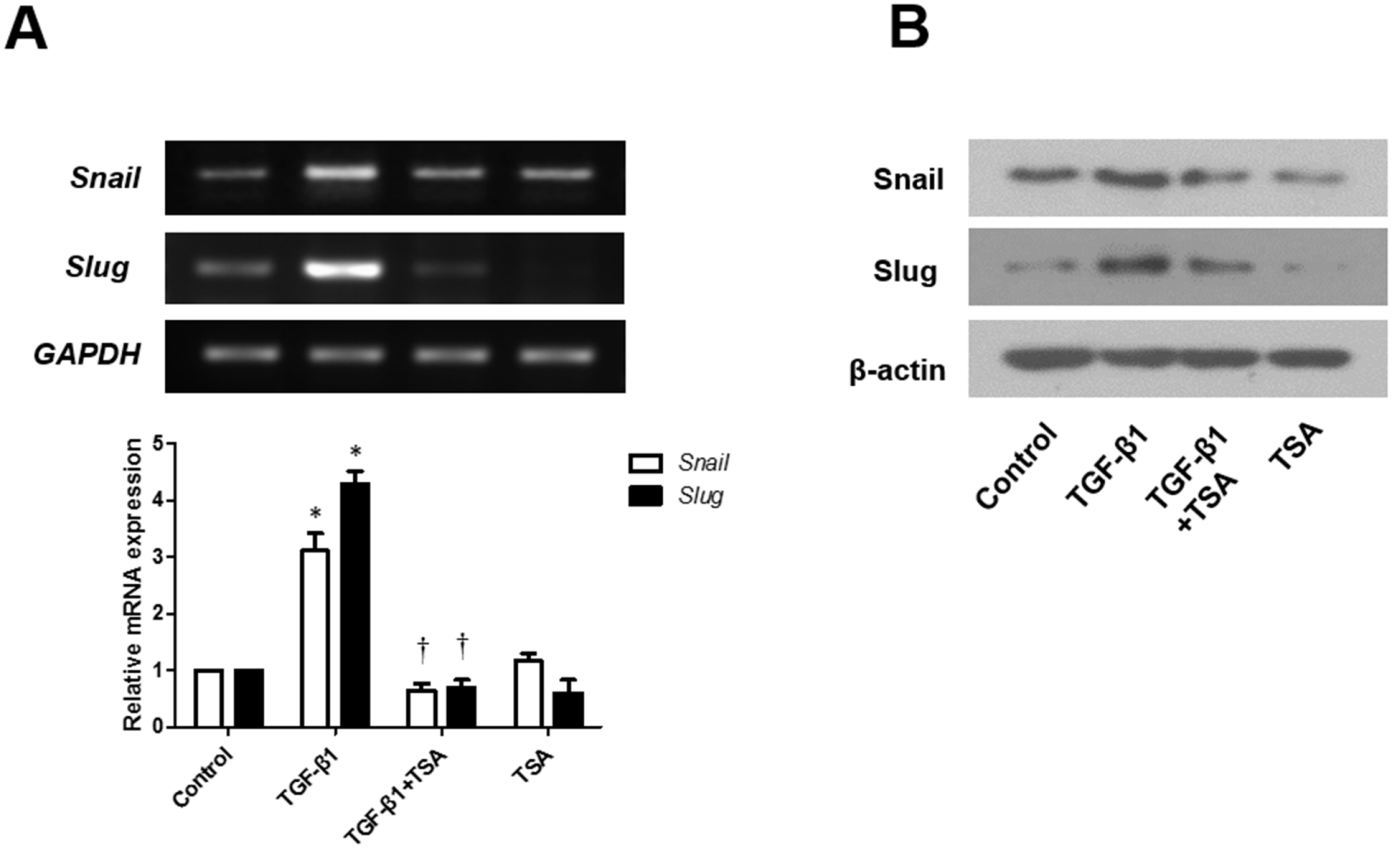
Effects of trichostatin A on expression of snail and slug mRNA and protein in TGF-β1-stimulated A549 cells were determined by RT-PCR (A) and western blotting (B) (Representative of independent experiments). Values are expressed as the mean ± standard error of the mean (SEM) of independent experiments. *P < 0.05 vs. control. †P < 0.05 vs. TGF-β1 alone. GAPDH, glyceraldehyde-3-phosphate dehydrogenase.

TGF-β1 induces EMT in primary airway epithelial cells [12]. To determine whether TGF-β1 induces EMT in A549 cells, cells were treated with 5 ng/mL of TGF-β1 for 48 h and change in their morphology was observed under a phase contrast microscope (Olympus japan, Dokyo, Japan). TGF-β1 treatment for 48 h resulted in the conversion from normal epithelial morphology with a cobblestone-like appearance into a migratory mesenchymal morphology with an abnormally elongated appearance. TGF-β1-stimulated A549 cells pretreated with TSA for 1 h returned to their normal epithelial morphology (Fig 2A). Expression of E-cadherin, vimentin, fibronectin, and α-SMA proteins as a marker of EMT was examined using western blotting and fluorescent immunocytochemical staining (Fig 2B and 2C). After treatment with TGF-β1 for 72 h, cells showed decreased E-cadherin and increased vimentin, fibronectin, and α-SMA expression. TSA pretreatment for 1 h inhibited the effects of TGF-β1 on EMT in A549 cells. As a last step in examining the inhibitory effect of TSA on EMT in TGF-β1-induced A549 cells, the level of EMT-related transcription factors such as snail and slug mRNA and protein was evaluated after 12 h for RT-PCR and 24 h for western blotting (Fig 3). TGF-β1 increased the mRNA and protein expression levels of slug and snail, and TSA pretreatment reversed the effect of TGF-β1.

### TSA inhibits the expression of HDAC2 and HDAC4 and induces acetylation of histone H3 and H4

**Fig 4:**
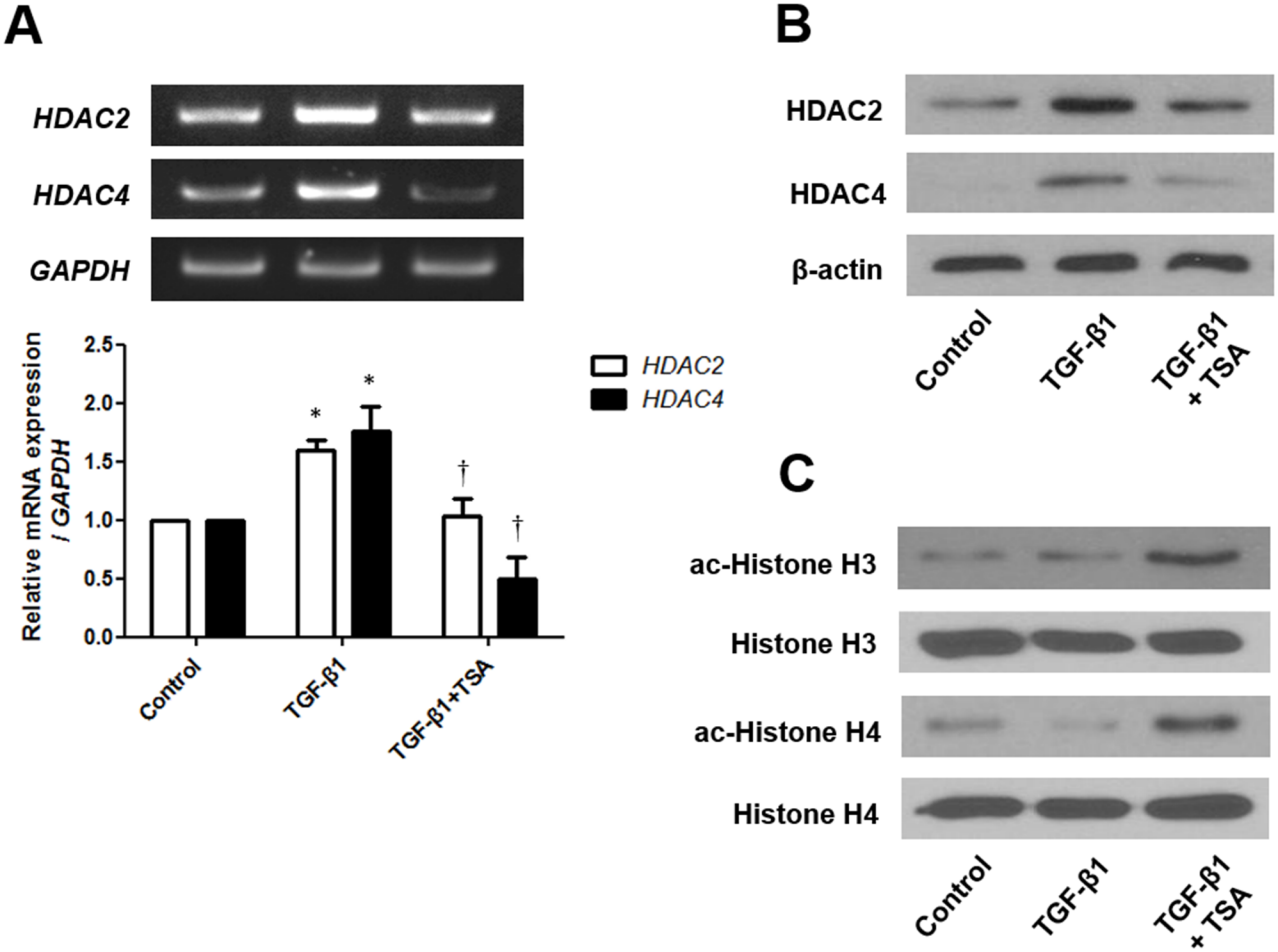
Effects of trichostatin A on expression of HDAC2 and HDAC4 mRNA and protein in TGF-β1-stimulated A549 cells were determined by RT-PCR (A) and western blotting (B) (representative of independent experiments). Effects of trichostatin A on hyperacetylation of histone H3 and H4 were determined by western blotting (C) (representative of independent experiments). Values are expressed as the mean ± SEM of independent experiments. *P < 0.05 vs. control. †P < 0.05 vs. TGF-β1 alone. GAPDH, glyceraldehyde-3-phosphate dehydrogenase.

TSA inhibits the activity of HDAC, leading to an increase in histone acetylation. Histone acetylation is related with the enhancement of specific genes. To determine inhibition of HDAC and hyperacetylation by TSA, the expression levels of HDAC2 and HDAC4 were measured by using RT-PCR and western blotting in A549 cells. TGF-β1 induced mRNA expression of HDAC2 and HDAC4 after 24 h, and HDAC2 and HDAC4 protein expression after 72 h. TSA pretreatment blocked the effects of TGF-β1 on HDAC2 and HDAC4 expression in A549 cells (Fig 4A and 4B). Next, we investigated acetylation of histone H3 and H4 with western blotting in A549 cells. TSA induces hyperacetylation of histone H3 and H4 after 72 h, regardless of TGF-β1 stimulation (Fig 4C). These results showed that TSA suppresses HDAC2 and HDAC4 and induces histone acetylation in A549 cells.

### Silencing of HDAC2 and HDAC4 enhances EMT in TGF-β1-induced A549 cells

**Fig 5:**
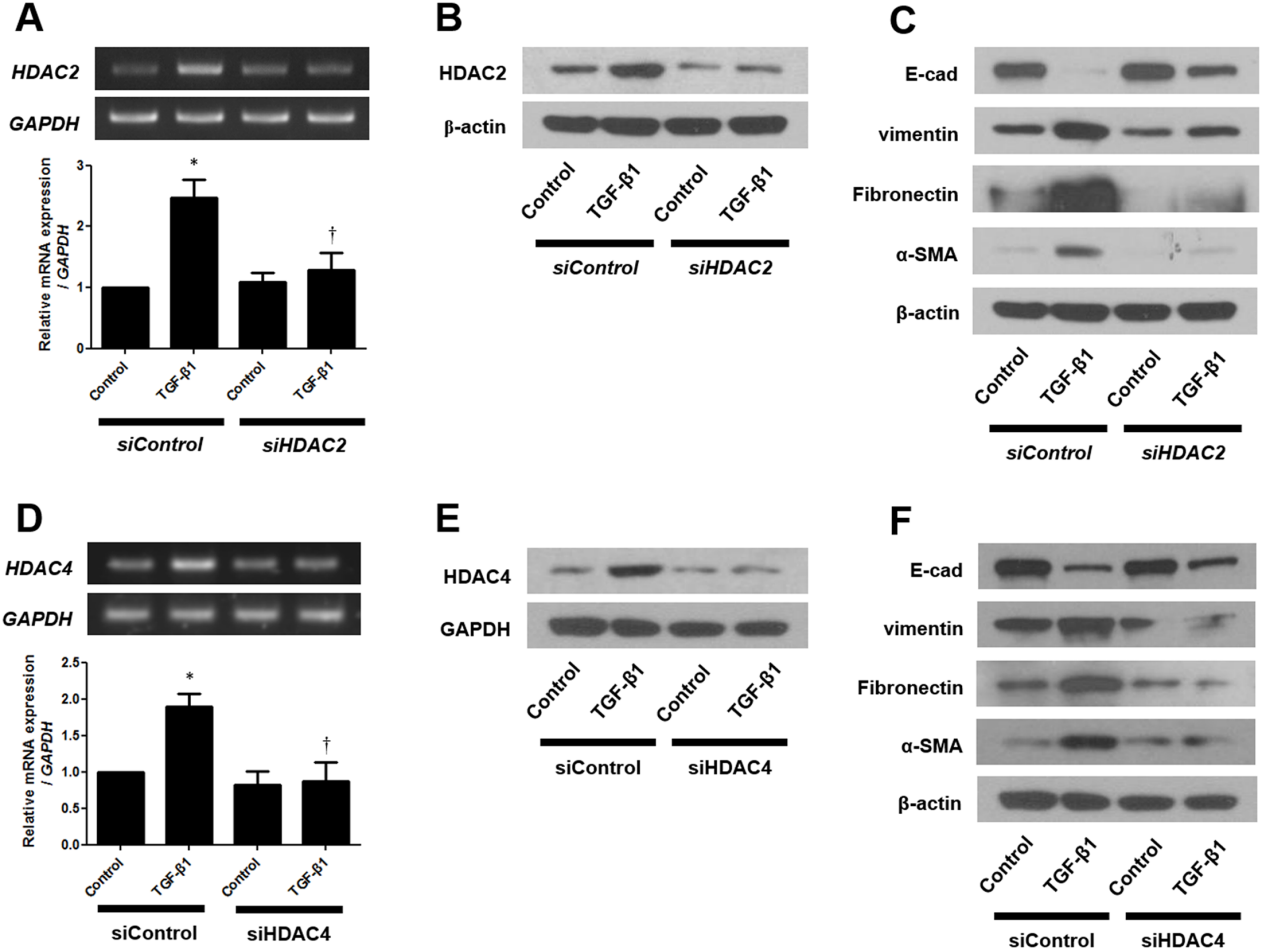
Effects of siHDACs on expression of HDAC2 and HDAC4 mRNA and protein in TGF-β1-stimulated A549 cells were determined by RT-PCR (A, D) and western blotting (B, E) (representative of independent experiments). Effects of siHDACs on expression of E-cadherin, vimentin, fibronectin, and α-smooth muscle actin protein in A549 cells were determined by western blotting (C, F) (representative of independent experiments). Values are expressed as the mean ± SEM of independent experiments. *P < 0.05 vs. control. †P < 0.05 vs. TGF-β1 alone. GAPDH, glyceraldehyde-3-phosphate dehydrogenase.

Next, the effects of silencing of HDAC2 by siRNA on TGF-β1-induced EMT were examined in A549 cells. After transfection of cells with siControl or siHDAC2, the mRNA and protein expression levels of HDAC2, E-cadherin, vimentin, fibronectin, and α-SMA were determined by RT-PCR and western blotting after 24 h and 72 h, respectively. In siHDAC2 pretreated cells, stimulation with TGF-β1 did not affect the expression levels of HDAC2, E-cadherin, vimentin, fibronectin, and α-SMA that were observed in siControl cells (Fig 5A, 5B and 5C). We also investigated the effects of silencing of HDAC4 in same manner. The results of silencing HDAC4 mirrored those of silencing HDAC2 (Fig 5D, 5E and 5F). These data indicated that epigenetic regulation by HDAC2 and HDAC4 is related with TGF-β1-stimulated EMT in A549 cells.

### TSA inhibits the migration of TGF-β1-induced A549 cells

**Fig 6:**
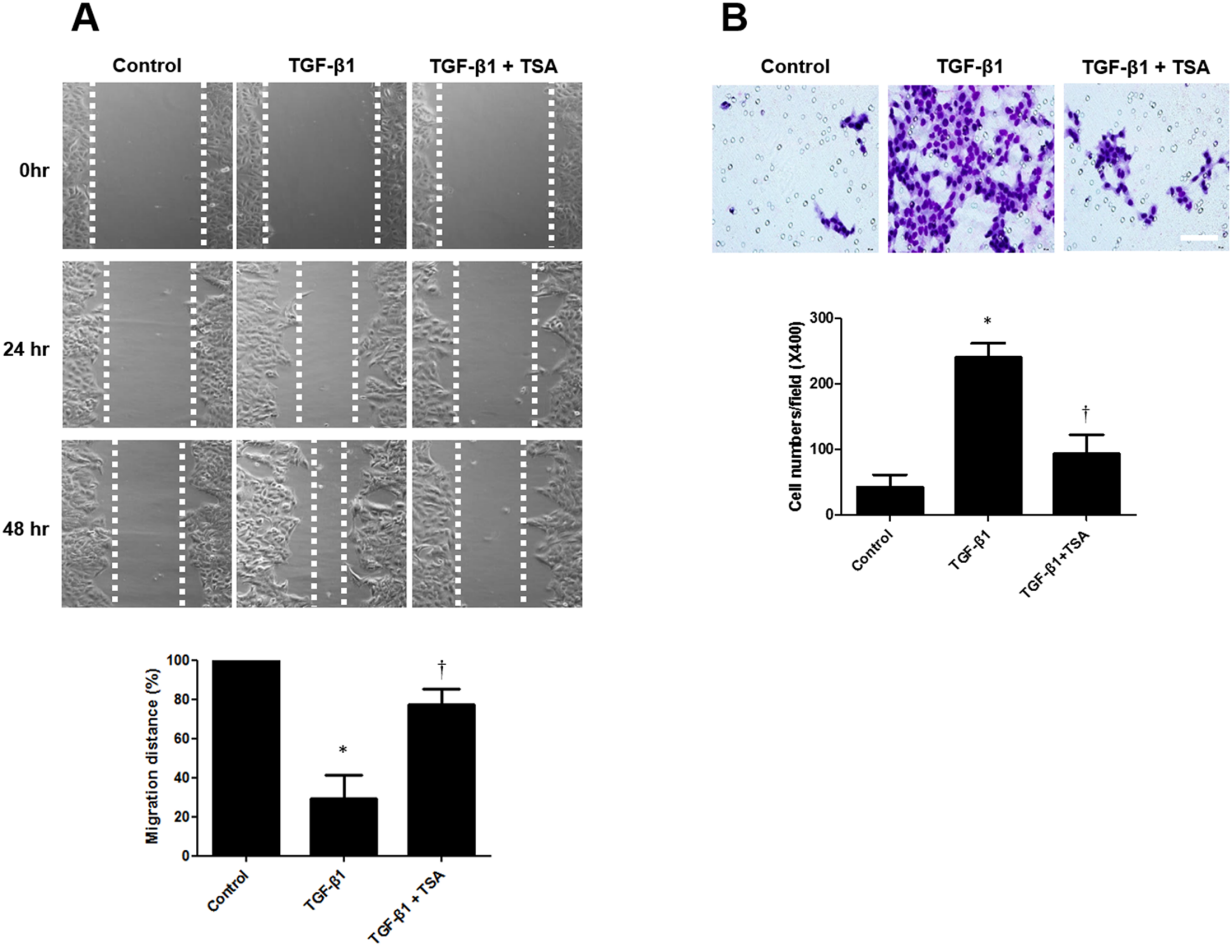
Effects of trichostatin A on migration ability of TGF-β1-stimulated A549 cells were measured using cell migration assay (A) and transwell invasion assay (B). Values are expressed as the mean ± SEM of independent experiments. *P < 0.05 vs. control. †P < 0.05 vs. TGF-β1 alone. Scale bar = 50 μm.

As increased migratory ability is a functional characteristic of mesenchymal cells, we assessed the change in migration capacity of A549 cells by using a cell migration assay. A straight scratch was made in adherent cells with a pipette tip. Then, we measured the distance that cells had migrated from the initial boundary after treatment with TGF-β1 with or without TSA. After 48 h, compared with the controls, cells migrated significantly further from the boundary of the initial wound area in TGF-β1-treated samples. However, pretreatment with TSA inhibited cell migration in TGF-β1-treated A549 cells (Fig 6A). To confirm the inhibitory effect of TSA on increased migratory ability of TGF-β1-induced A549 cells, we performed a transwell invasion assay. After treatment with TGF-β1 with or without TSA for 48 h, we counted the number of cells that had spread through the filter and adhered to the underside. The results from the transwell invasion assay also showed that pretreatment with TSA blocks the increased cell invasion in TGF-β1-treated cells (Fig 6B).

### TSA inhibits TGF-β1-induced EMT in PNACs and organ culture

**Fig 7:**
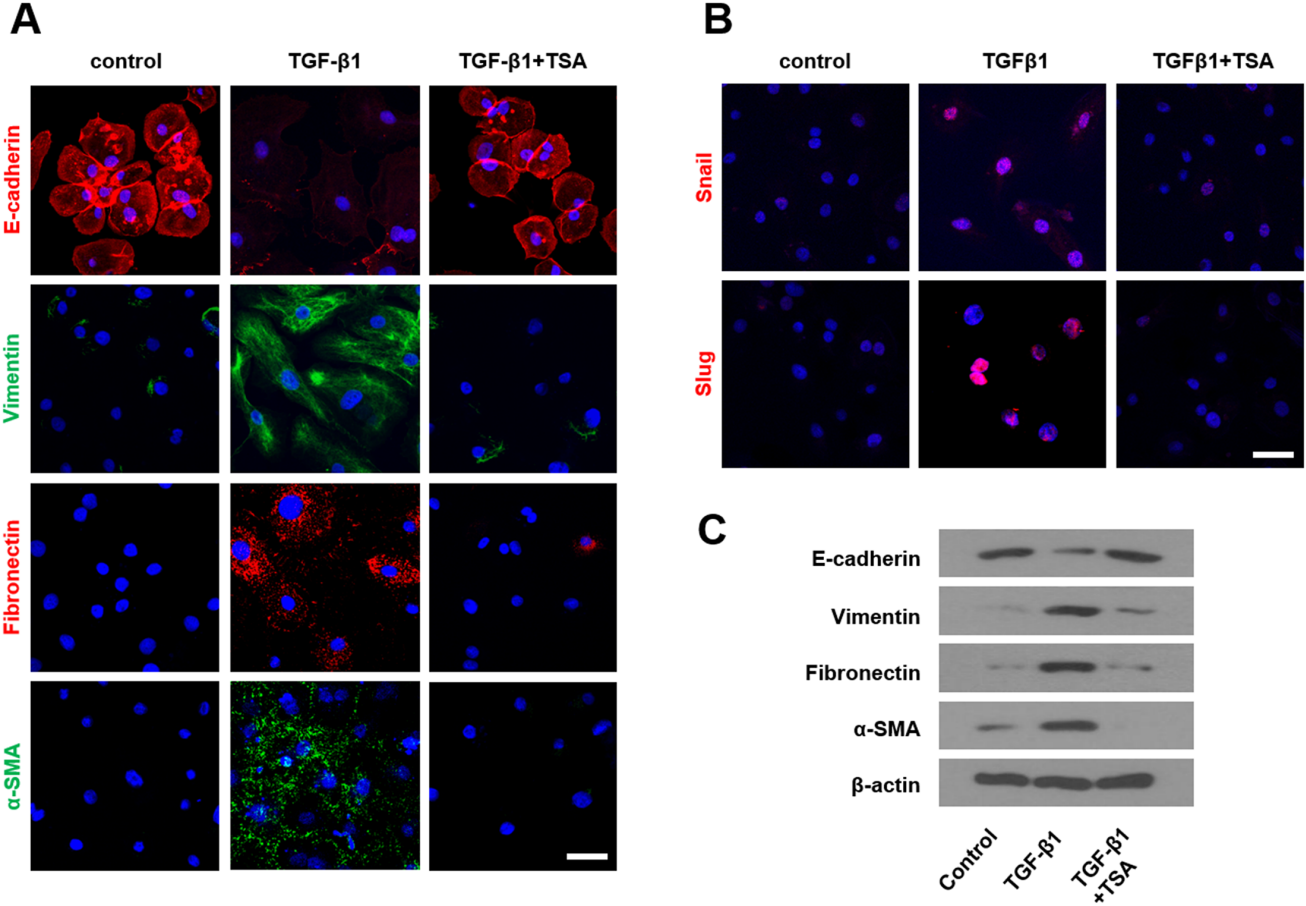
Effects of trichostatin A on expression of E-cadherin, vimentin, fibronectin, α-smooth muscle actin, snail, and slug proteins in TGF-β1-stimulated primary nasal epithelial cells were determined by immunofluorescent staining (A). Effects of trichostatin A on expression of E-cadherin, vimentin, fibronectin, and α-smooth muscle actin protein in TGF-β1-stimulated inferior turbinate tissue were determined by western blotting (B). Representative of independent experiments. Scale bar = 50 μm.

To assess whether the inhibitory effects of TSA on TGF-β1-induced EMT in A549 cells are also seen in nasal tissue, we repeated several experiments in primary PNECs and in inferior turbinate organ culture. To determine whether TGF-β1 induces EMT in PNECs, we treated cells with 5 ng/mL of TGF-β1 for 72 h and observed E-cadherin, vimentin, fibronectin, and α-SMA protein expression using a fluorescence microscope. To determine protein expression of snail and slug, we treated cells with TGF-β1 for 24 h. After the treatment with TGF-β1, cells showed decreased E-cadherin and increased vimentin, fibronectin, α-SMA, snail, and slug expression. TSA pretreatment for 1 h inhibited the effects of TGF-β1 on EMT in PNECs (Fig 7A and 7B). To identify whether EMT is induced by TGF-β1 and inhibited by TSA in nasal inferior turbinate organ cultures, organ cultures were exposed to TGF-β1 for 72 h with or without TSA, and were checked for E-cadherin, vimentin, fibronectin, and α-SMA protein expression levels using western blotting (Fig 7C). Expression levels of vimentin, fibronectin, and α-SMA were increased and E-cadherin expression level was decreased in TGF-β1-treated inferior turbinate organ cultures compared with the control. Pretreatment with TSA reversed the effect of TGF-β1 on all mentioned EMT markers. These results indicate that TSA also suppresses EMT induced by TGF-β1 in nasal cells and tissue.

## Discussion

The present study showed that TSA inhibits TGF-β1-induced EMT in A549 cells, PNECs, and inferior turbinate organ culture. TGF-β1 altered the mRNA and protein expression levels of EMT markers including E-cadherin, vimentin, fibronectin, α-SMA, slug, and snail, and pretreatment with TSA reversed the effect of TGF-β1. TSA inhibited the expression of HDAC2 and HDAC4, and induced histone acetylation in A549 cells. Silencing of HDAC2 and HDAC4 by siRNA enhanced TGF-β1-induced EMT in A549 cells. When we investigated the migratory ability of A549 cells after TGF-β1 stimulation via cell migration assay and transwell invasion assay, we found that they migrated significantly further than the control. However, TSA blocked the effect of TGF-β1 on the migratory ability of cells. In the experiments using PNECs and inferior turbinate tissue, TSA suppressed the expression of EMT markers induced by TGF-β1.

Remodeling is an important feature of wound healing. It is a dynamic process involving matrix production and degradation in response to inflammatory insult. Tissue remodeling can lead to a normal reconstruction process [14]. However, when remodeling causes many alterations in the composition, content, and organization of constituents of the organs, thereby causing morphological or functional disabilities, it can be considered pathological [15]. As in numerous other chronic inflammatory diseases, CRS patients develop persistent chronic inflammation of the mucosa. In a study examining histological specimens from 22 patients with refractory CRS undergoing endoscopic sinus surgery, epithelial damage such as epithelial shedding and basement membrane thickening was observed in all cases [16]. Because such remodeling processes are somewhat irreversible, there is a growing consensus that functional endoscopic sinus surgery that targets restoration of function by improving ventilation and allowing mucociliary clearance to normalize is not perfect answer to the refractory CRS [17].

Prevention of EMT is now considered an effective measure for the inhibition of tissue remodeling. Airway epithelium is a barrier between the host and the environment, and represents the first line of defense against microorganisms and allergens [18]. Airway epithelium acts as a physical barrier and is composed of apical tight junctions and underlying adherens junctions [19]. It is now recognized that airway epithelium attends a variety of immunological mechanisms by releasing cytokines and interacting with immune cells. EMT is a process essential in wound healing and tissue remodeling after injury [20]. However, in an unsuccessful attempt to repair the injured tissue that is can be happened in constant damage caused by chronic inflammation, EMT can lead to the destruction of the functions of the epithelium as a physical barrier and immune regulator. For this reason, EMT is a novel clinical therapeutic target in many chronic airway diseases. In fact, EMT was observed in several chronic inflammatory airway diseases, including asthma, COPD, and bronchiolitis obliterans syndrome [13,21,22]. There is also evidence demonstrating that epithelial cells express mesenchymal markers in CRS. We supposed that functional loss of airway epithelium caused by EMT is one of the main reasons for the unresponsiveness of recalcitrant CRS to maximal medical and surgical treatment.

Epigenetic changes are changes in gene expression that do not alter the underlying DNA sequence. DNA methylation and histone modification are the most well-known mechanisms of epigenetics [23]. Histone acetylation, which is regulated by histone acetyltransferase and histone deacetylase, is one type of chromatin modification [24]. Acetylation of histones relaxes nucleosomes, thereby activating gene induction. On the contrary, histone deacetylase induces gene silencing by removal of acetyl groups from histones. Imbalance between the activities of HATs and HDACs can lead to disease states [25]. For this reason, TSA, which inhibits HDACs in a noncompetitive and reversible way, has been studied in various diseases, including cancer and fibrosis. Evidence has shown that the anti-fibrotic and anti-cancer effects of TSA are related with EMT. Wang et al. [26] showed that TSA reverses EMT in colorectal cancer cells and prostate cancer cells thereby explaining that TSA suppresses invasion and migration of cancer cells. In a study with renal cells and hepatocytes, TSA exerted anti-EMT effects[27,28]. Related with CRS, we have previously shown that HDAC2 is elevated in nasal polyps, suggesting that they may serve as potential targets of treatment and that TSA inhibits extracellular matrix production in nasal polyps [29,30]. Based on the above evidence, we can draw a hypothesis that HDAC inhibition by TSA is associated with EMT in airway epithelial cells.

In conclusion, we demonstrated that EMT is induced by TGF-β1 in airway epithelial cells and nasal tissue via activation of HDAC2 and HDAC4, and that inhibition of HDAC2 and HDAC4 by TSA reduces TGF-β1-induced EMT. This observation indicates that histone deacetylase inhibitors such as TSA could be considered as candidates for treatment of recalcitrant CRS related with tissue remodeling.
